# Severe thyrotoxicosis in an infant revealing familial nonautoimmune hyperthyroidism with a novel (C672W) stimulating thyrotropin receptor germline mutation

**DOI:** 10.1002/ccr3.1178

**Published:** 2017-10-25

**Authors:** Isabelle Oliver‐Petit, Frédérique Savagner, Solange Grunenwald, Magaly Vialon, Thomas Edouard, Philippe Caron

**Affiliations:** ^1^ Endocrine, Genetics, Bone Diseases, and Paediatric Gynecology Unit Children's Hospital CHU Toulouse Toulouse France; ^2^ Biochemistry and Genetic laboratory Federative institute of biology CHU Purpan Toulouse Toulouse France; ^3^ Department of Endocrinology and Metabolic Diseases Cardio‐Vascular and Metabolic Unit CHU Larrey Toulouse France

**Keywords:** Familial nonautoimmune hyperthyroidism, iodine intake, mitral valve prolapse, proptosis, stimulating TSH receptor gene mutation

## Abstract

We describe severe thyrotoxicosis in young members of a family with nonautoimmune hyperthyroidism caused by a C672W germline mutation in exon 10 of TSHR gene. In this family, lack of genotype‐phenotype correlation and anticipation across generations could be linked to an increased iodine intake as recently observed in France.

## Introduction

TSH receptor is a plasma‐membrane G protein‐coupled receptor and consists of seven transmembrane domains (TDMs), a large extracellular domain with binding site for TSH and a small intracytoplasmic tail. TSH receptor mediates the effect of TSH in thyroid development, growth and hormones synthesis.

The TSH receptor gene is encoded by 10 exons on chromosome 14q31. Most activating germline mutations of the TSH receptor gene are located in the cytoplasmic loops and transmembrane domains, and cause nonautoimmune sporadic congenital hyperthyroidism or familial nonautoimmune hyperthyroidism. Since the first case of familial nonautoimmune hyperthyroidism published in 1982 1, at least 21 different mutations of the TSH receptor gene have been identified as a cause of autosomal dominant nonautoimmune hyperthyroidism in 28 families with more than 122 affected individuals 2, 3, 4, 5, 6, 7, 8, 9, 10, 11, 12.

Patients with nonautoimmune hyperthyroidism present with classical signs and symptoms of thyrotoxicosis, but the severity is variable among patients from families with different TSH receptor gene mutations and even among affected patients belonging to the same family and harboring the same mutation 2, 4, 5, 8, 13.

In this article, we describe severe thyrotoxicosis in an infant revealing a familial nonautoimmune hyperthyroidism with novel C672W stimulating TSH receptor germline mutation. Then, we discuss clinical characteristics of the C672W family in the context of reported literature.

## Patients and Methods

### Patients

**Figure 1:**
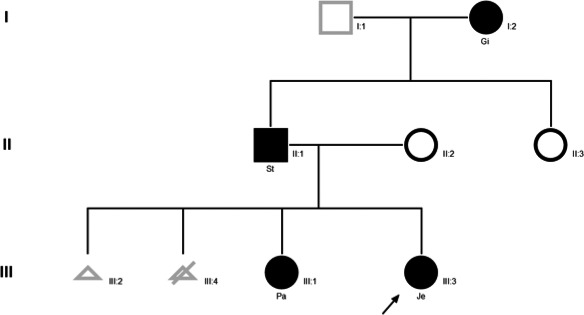
Pedigree of the C762W family. The arrow identifies the index patient. Solid square and circles indicate members with hyperthyroidism.

The pedigree of the family is shown in Figure 1.

**Figure 2:**
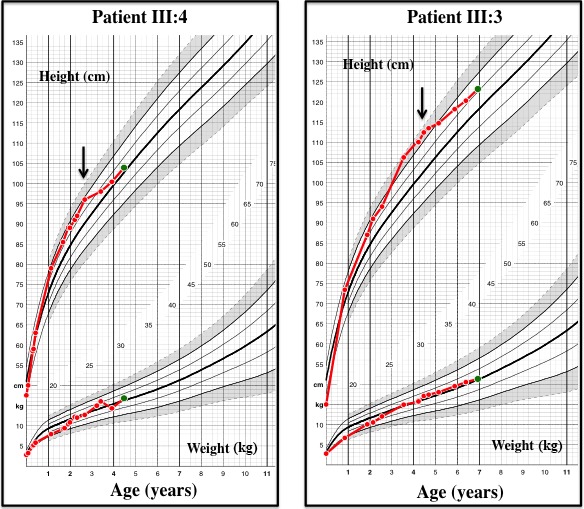
Patient III:4 and III:3 growth charts for height and weight from birth to diagnosis (on French girls references for age), showing failure to thrive for weight and growth advance. The arrow indicates time of diagnosis and beginning of treatment.

**Figure 3:**
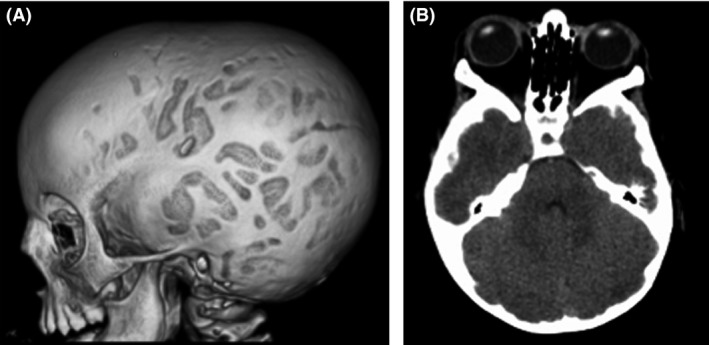
Radiologic data of the index patient (III:4) (A) craniosynostosis with digital impressions on cranial three‐dimensional CT scan. (B) CT scan at 20 months showing bilateral proptosis of grade 2 secondary to the craniosynostosis.

The index patient (III:4), second daughter of unrelated parents, was delivered by cesarean section at 35 weeks of gestational age for fetal tachycardia (185/min). Her birthweight was 2800 g (0.7 SDS), birth length 47.5 cm (0.3 SDS), head circumference 32 cm (−0.1 SDS), and Apgar score 10/10 at 1 and 5 min. She was referred to the hospital at 20 months of age for tachycardia (180–200/min). This symptom had yet been noticed at 6 weeks and 5 months when she was briefly hospitalized for hyperthermia or diarrhea. Parents reported day and night psychomotor agitation and overeating from first weeks of life. She presented growth advance for height 85.5 cm (+1.9 SDS for national French references), low weight 9.180 kg (−1.6 SDS) (Fig. 2), BMI 12.7 kg/m^2^ (<−2 SDS), and head circumference 45.5 cm (−1.5 SDS) with failure to thrive. Bone age was advanced to 5 years. She presented bilateral proptosis (at Hertel's exophthalmometer) without inflammatory features. Three‐dimensional CT scan showed digital impressions on cranial vault (Fig. 3A), and CT scan showed grade 2 proptosis due to less prominent external wall of the orbit secondary to craniosynostosis (Fig. 3B). Examination confirmed tachycardia and cardiac ultrasonography identified mitral valve prolapse with minor mitral insufficiency. She had a goiter and neck ultrasonography showed diffuse enlargement of the thyroid gland (volume: 4 cm^3^, normal for age <2.5 cm^3^) with high blood flow. Thyroid function tests confirmed severe thyrotoxicosis [free T3 > 20 pg/mL (normal range: 2–4.3 pg/mL), free T4: 52 pg/mL (9.3–17 pg/mL) and TSH: 0.005 *μ*U/mL (0.27–4.2 μU/mL)] without anti‐TSH receptor antibody.

A treatment with propranolol (2 mg/kg/day) and thiamazole (0.6 mg/kg/day) was initiated. Psychomotor restlessness and hypotonia were quickly resolved, but hyperthyroidism was only partially reduced after 3 weeks (free T4: 33 pg/mL and free T3: 9.8 pg/mL): Thiamazole dose was increased to 1 mg/kg/day. After 3 months, thyroid hormones levels were at the upper limit of normal range, and propranolol treatment was withdrawn (Table 1). On ultrasonography, thyroid enlargement and high blood flow in thyroid tissue were not reduced after 1‐year treatment. Total thyroidectomy was performed at 34 months of age, complicated by bilateral transient recurrent palsy. During postoperative follow‐up, the patient was euthyroid with levothyroxine substitutive therapy.

**Table 1:** Changes of thyroid function parameters (free T4, free T3, TSH) according to thiamazole treatment during follow‐up of patients 1 (III:3) and 2 (III:1) with stimulating C672W TSH receptor gene mutation

	Day 0	Day 21	Day 75	Day 100	Month 6	Month 9	Month 12
Patient 1							
Free T3 pg/mL (2–4.2)	>20	9.8	6.1	4.9	5	4.5	5
Free T4 pg/mL (7.5–16)	52	33.3	27.8	37.1	19.8	17.2	15.8
TSH mU/L (0.4–4.4)	0.005	0.009	<0.005	<0.005	0.005	0.005	0.005
Thiamazole mg/kg/day	0.55	1	0.9	0.9	1	1	1
Patient 2							
Free T3 pg/mL (2–4.2)	15.3	7.6	4.39	5.9	2.5	2.72	3.1
Free T4 pg/mL (7.5–16)	39.9	30.2	24.3	20.9	7	10.5	7.1
TSH mU/L (0.4–4.4)	0.006	0.009	<0.005	<0.005	0.1	0.32	0.57
Thiamazole mg/kg/day	0.65	0.65	0.6	0.75	0.75	0.75	0.75

Patient 2 (III:3) is the older sister, born at 39 weeks of gestation with birthweight 2780 g (−1.5 SDS), birth length 45 cm (−2.5 SDS), and head circumference 32 cm (−2.5 SDS). She was the first child because her mother had previously two pregnancies ended by two miscarriages. Because the diagnosis of hyperthyroidism of her sister, she was referred to hospital at 4 years and 2 months. She presented advanced growth and hypotrophy with height 110 cm (+2 SDS), weight 15.6 kg (0 SDS), BMI 15.6 kg/m2 (<−2 SDS), and head circumference 46.5 cm (−2.2 SDS) (Fig. 2). Bone age was advanced to 8 years, and cranial X‐ray showed digital impressions and craniosynostosis. Physical examination showed a small goiter, tachycardia (136/min), and bilateral proptosis (19 mm) without inflammatory signs. Ultrasonography of the neck confirmed thyroid enlargement for age (thyroid volume 5.5 cm^3^; normal for age <3 cm^3^) with high blood flow, and cardiac ultrasonography identified mitral valve prolapse.

Thyroid hormones test showed thyrotoxicosis (free T3: 15.3 pg/mL, free T4: 40 pg/mL, TSH: 0.006 μU/mL). Thiamazole therapy was initiated (0.6 mg/kg/day), and euthyroid status was obtained after 8 weeks (Table 1). Total thyroidectomy was performed after 1 year of antithyroid drug treatment. She is euthyroid with levothyroxine substitutive therapy.

The girls' father (II:1) had been seen in pediatric department at 8 years of age for growth advance with pectus excavatus, genu varum, cryptorchidism, and high‐arched palate. Cardiac examination revealed mitral valve prolapse. Hyperthyroidism was diagnosed at 17‐year‐old, and treated with antithyroid drug during 10 years. At the age of 27 years, he presented multi‐nodular and compressive goiter, and total thyroidectomy was performed. Histologic examination revealed bifocal papillary carcinoma (0.7 and 1.5 cm). The patient received ^131^I treatment (100 mCi) and substitutive therapy with levothyroxine (TSH: 0.46 mU/L). During follow‐up, echocardiography shows mitral valve insufficiency, and thyroglobulin concentration was <0.2 ng/mL and cervical echography was normal.

**Figure 4:**
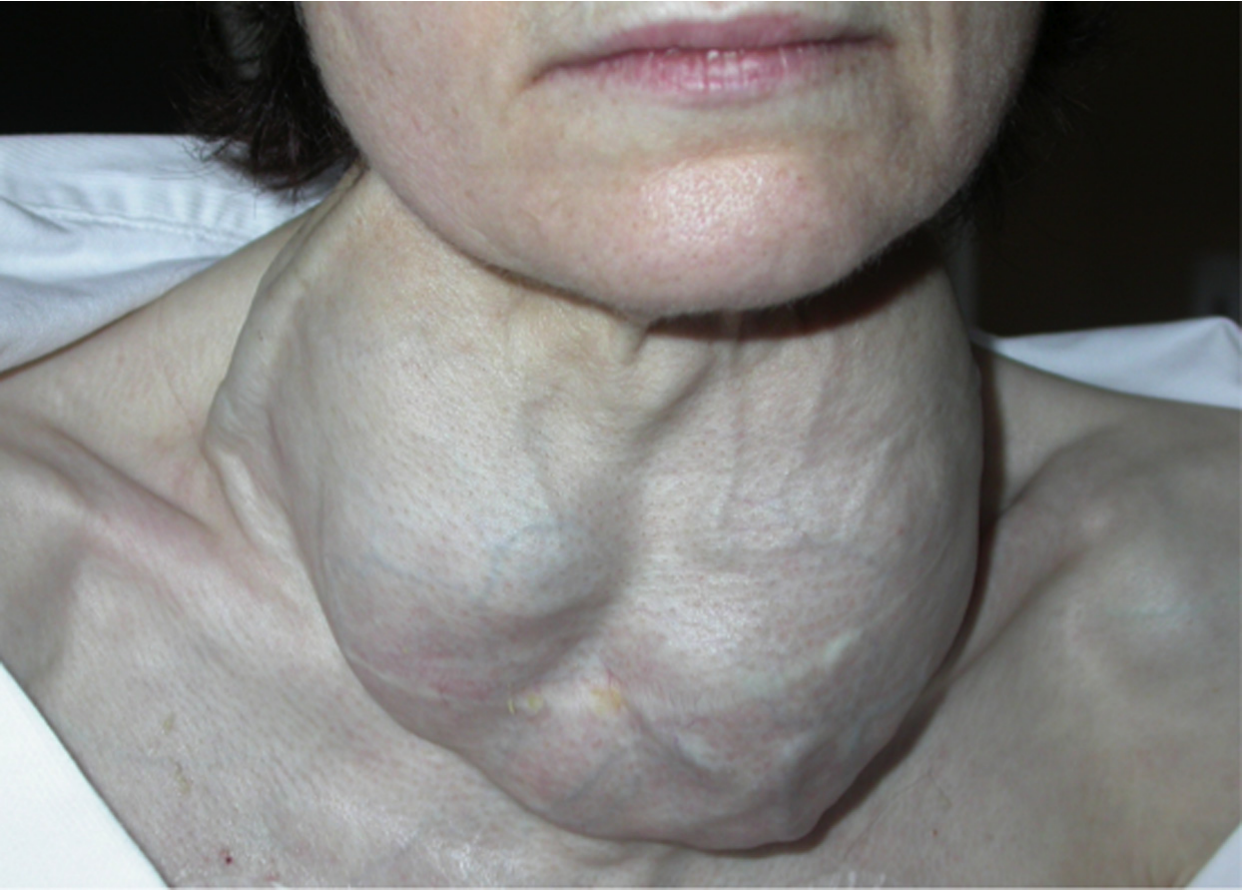
Multinodular and vascular goiter of patient 4 (I:2).

The father's mother (I:2) was diagnosed with hyperthyroid goiter at 27 years of age, and she had partial thyroidectomy. Due to recurrence of hyperthyroid symptoms, she was treated with antithyroid drug without regular endocrine monitoring. At the age of 56 years, the treatment was 20 mg carbimazole and she presented huge, multinodular vascular goiter (Fig. 4) and tachyarrhythmia with congestive heart failure. Thyroid function tests confirmed thyrotoxicosis with high free T4 (22.8 pg/mL) and free T3 (12 pg/mL) levels without anti‐TSH receptor antibody. Cardiac echography revealed organic mitral insufficiency. Antithyroid drug treatment was changed to 1200 mg propylthiouracyl, and the patient had total thyroidectomy for benign multinodular goiter. She presented postoperative transient left recurrent palsy, and she had then normal thyroid function on low‐dose levothyroxine therapy.

All adult patients have been informed, and patient's agreements were obtained for this study.

### Methods

DNA was extracted from peripheral blood leukocytes from the index patient, her sister, her parents, her paternal grandmother, and from carcinomatous thyroid cells of the girl's father (Qiagen Blood kit, QIAGEN, Hilden, Germany). Amplification of all the coding sequences of the TSHR gene (NM_000369.2) was performed by PCR using selected primers referred as Exon 10 sense: 5′‐GCTATGCCAAAGTCAGTATC‐3′ and reverse: 5′‐TAAGTTCCCCTACCATTGTG‐3′. Samples were tested using BigDye terminator chemistry according to the manufacturer's instructions and analyzed onto an ABI 3100 sequencer (all from Applied Biosystems, Foster City, CA, USA).

FACS analyses were realized on transfected cells incubated 30 min with 1/400 of a mouse anti‐hTSHR antibody (2C11, Abcam, Cambridge, UK) followed by an incubation with 1/400 dilution of an Alexa Fluor 488‐labeled goat antimouse IgG (Invitrogen, Carlsbad, CA, USA) in FACS buffer. Receptor expression was determined by the median fluorescence intensity obtained during FACS analysis (FACscan, Beckton Dockinson, Franklin Lakes, NJ, USA) and reported to 100 wild‐type hTSHR expression.

Functional analysis related to cAMP production was performed into COS‐7 cells after transient transfection with wild‐type (WT) or C672W mutations 2. Mutations were introduced into hTSHR‐pCMV6 plasmid (Origene, Rockville, MA, USA) using site‐directed mutagenesis with specifically designed primers and according to manufacturer's protocol (QuickChange^®^ Site‐Directed Mutagenesis kit, Stratagene). COS‐7 cells were transfected with 1 μg of WT or mutant (C672W) and 0.5 μg of luciferase reporter vector containing cAMP response element (pGL4.29, Promega, Madison, WI, USA). For Gq/11 activation, COS‐7 cells were cotransfected with TSHR cDNAs and a reporter construction containing the luciferase gene under the control of NF‐TA (reference 10959, Addgene). Forty‐eight hours after transfection, cells were incubated in DMEM with or without bovine TSH (100 mU/mL) for 30 min (Sigma, St Louis, MO, USA). Luciferase activity was normalized to β‐galactosidase. Gs*α* and Gq/11 effects were reported by respective luciferase activities using luciferase reporter gene assay according to the manufacturer's instructions (Promega). All transfections were performed in triplicate, and the experiments were repeated three times.

## Results

In the index case, her sister, father, and paternal grandmother, a heterozygous c. 2016 T>G mutation was identified in the exon 10 of the TSHR gene. Molecular analysis identified the same heterogenous c.2016 T>G mutation in DNA extracted from carcinomatous thyroid cells of the girl's father. As a result, cysteine at the codon 672 was changed to tryptophan (p.Cys672Trp or C672W) in the 7th transmembrane domain of the TSH receptor.

**Figure 5:**
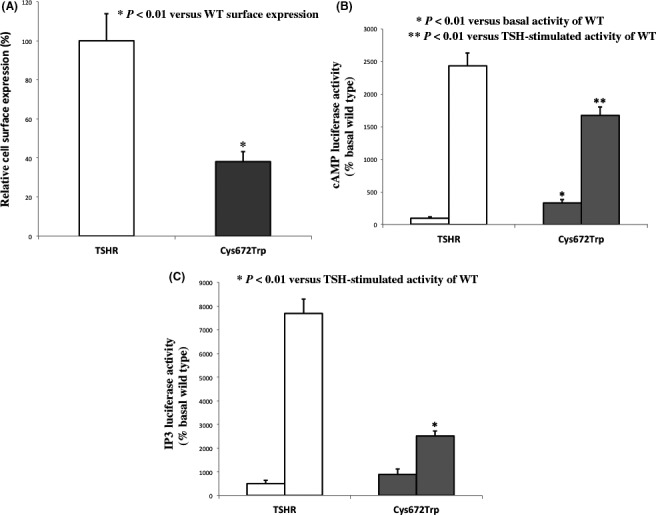
Characterization of the C672W mutation: (A) Cell surface expression of wild type and C672W mutant (B) Activation of adenylate cyclase 48 h after transfection. Basal activity of wild type was set at 100%. Cells were stimulated with bovine TSH. Data represent the mean ± SEM. (C) Activation of phospholipase Cβ pathway 48 h after transfection. Basal activity of wild type was set at 100%. Cells were stimulated with bovine TSH. Data represent the mean ± SEM.

The characterization of the C672W mutation revealed a 38% reduced cell surface expression of the receptor compared to wild‐type TSHR (Fig. 5A). The basal activity of C672W mutation was significantly increased compared to the wild type (331 ± 55%, *P* < 0.01). When stimulated with bovine TSH, the cAMP response of C672W mutant was significantly reduced compared to wild type (WT: 2435 ± 119%, C672W: 1674 ± 203%, *P* < 0.01) (Fig. 5B). Determination of basal and TSH‐induced activation of the Gq/11 signaling pathway revealed that basal activity was slightly increased compared to the wild type (NS), whereas a threefold decreased activation of the C672W mutant (32 ± 11%) was observed during TSH activation (Fig. 5C).

## Discussion

Most stimulating germline mutations of TSH receptor have been identified in exon 10 of the receptor gene encoding for the transmembrane and intracellular portions of the TSH receptor. In this family, the C672W mutation is present in the 7th transmembrane segment of the TSH receptor. A previous p.Cys672Tyr (C672Y) mutation has been identified in familial nonautoimmune hyperthyroidism 2, underlying the role of this cysteine residue in the TSHR function. In cells expressing the mutated (C672W) receptor, the basal cytoplasmic cAMP level is higher than in cells transfected with wild type, so that stimulating mutation of the TSH receptor gene cause constitutive activation of the intracellular signaling cascade (Gs/adenylyl cyclase system) inducing thyroid growth and thyroid hormone synthesis. These results are similar to that observed for previous C672Y mutation 2.

In familial nonautoimmune hyperthyroidism, age at diagnosis and severity of the thyrotoxicosis are highly variable depending in part on the intensity of the activating mutation allele 2. In this family, the C672W mutation was associated with early signs of thyrotoxicosis, the younger infant presented tachycardia from the fetal and neonate period, and severe thyrotoxicosis and goiter in all affected patients. The lack of genotype–phenotype correlation in patients with familial nonautoimmune hyperthyroidism has been reported and might reflect the influence of other genetic, epigenetic, or environmental factors 4, 6, 8, 14. Anticipation across generation has already been reported and a general tendency toward a younger age presentation of the disease in subsequent generations could be linked to environmental factors 4, 6, 8. In the reported family, the genotype alone could not explain the variable phenotype presentation of affected patients with the C672W mutation. During the second half of 20th‐century SUVIMAX survey reported that French population was exposed to mild iodine deficiency 15, whereas in recent ENNS survey, the iodine status was considered as satisfactory in adult population 16. Thus, France is no more considered as a country at risk of iodine deficiency 17. Beside the constitutive activation of the cAMP pathway secondary to the stimulating mutation of the TSH receptor gene, increase in iodine intake could therefore influence the age of diagnosis and the severity of the thyrotoxicosis presented by the affected members of the family.

Maternal and fetal hyperthyroidism may severely increase the risk of complications during pregnancy such as fetal loss, preeclampsia, low birthweight, preterm delivery, and stillbirth. In the C762W family, the mother has presented two pregnancies ended in spontaneous miscarriages (Fig. 1). Considering thyroid disorders due to mutations of TSH receptor gene, enhanced human chorionic gonadotropin (hCG) sensitivity of mutant TSH receptor can cause severe gestational thyrotoxicosis and unexplained multiples abortions as recently reported in one family 18. Stimulating TSH receptor germline mutation can also cause early fetal hyperthyroidism leading to fetal death and abortion 19. Therefore, miscarriages observed in this family could be related to fetal and severe hyperthyroidism in the context of C672W TSH receptor gene mutation under the probable influence of environmental factors (iodine intake).

Absence of ocular symptom in the first families with nonautoimmune congenital hyperthyroidism was not confirmed in more recent publications. In the C672W family, the two sisters presented bilateral proptosis without inflammatory features, and an axial CT scan of index patient shows a bilateral proptosis secondary to craniosynostosis without any enlargement of ocular muscles. Another explanation of ocular symptoms could be the activation of the TSH receptor on orbital fibroblasts and adipocytes, but adult patients (II.1 and I.2) do not present proptosis or exophthalmia.

Mitral valve prolapse in patients with familial nonautoimmune hyperthyroidism has been previously associated with P639S (Pro639Ser) mutation of the 6th transmembrane domain of the TSH receptor in a Chinese family with severe thyrotoxicosis 3. All hyperthyroid members of the C672W family presented mitral valve prolapse or insufficiency suggesting that this association is not fortuitous, and that severe and early thyrotoxicosis itself is the main pathogenic factor of the mitral valve prolapse and insufficiency observed in those patients. TSH receptor is expressed in the heart, and Gq activation could induce intracellular calcium mobilization 20, 21. However, this mechanism was not demonstrated for C672W. TSH receptor activation might increase the clinical expression of mitral valve prolapse in genetically predisposed individuals 3, as well as severe and early thyrotoxicosis could cause papillary muscle dysfunction and be responsible of mitral valve prolapse in young hyperthyroid patients 22.

In sixty‐seven operated patients with familial nonautoimmune hyperthyroidism reported in the literature, two had micropapillary 1 and one oncocytic 7 carcinomas. Histology of father's thyroid revealed bifocal papillary carcinoma, and the patient was cured by surgery and radioiodine therapy. Despite early and unregulated stimulation of G protein pathway in thyroid cells, the small number of differentiated thyroid carcinoma in patients with stimulating TSH receptor gene mutation is consistent with their low frequency in other thyroid diseases with chronic TSH receptor activation 23.

All affected members of C672W family were primarily treated with surgery. However, in hyperthyroid patients with stimulating TSH receptor mutation, surgery is rarely definitively curative as observed in the grandmother of this family. In the context of possible complications of thyroid surgery (recurrent palsy, hypoparathyroidism) and the frequent relapse of thyrotoxicosis, a radioiodine treatment could be an alternative for young patients with thyrotoxicosis and homogenous small goiter 24. According to 2012 ETA guidelines, radioiodine treatment is recommended only in children over 5 years. In the C672W family, long‐term clinical and hormonal follow‐up of the pediatric patients is required as there is likelihood of relapse with the indication of definitive treatment by radioactive iodine.

## Authorship

IO‐P: contributed to the study hypothesis, data collection and analysis, and wrote the manuscript and shared scientific discussions. FS: contributed to the study hypothesis, performed all molecular studies, and wrote the manuscript and shared scientific discussions. SG: contributed to data collection and analysis from adult patients, and revised the manuscript. MV: contributed to data collection and analysis from pediatric patients, and shared scientific discussions. TE: contributed to data collection and analysis from pediatric patients, shared scientific discussions, and revised the manuscript. PC: contributed to the study hypothesis, data collection and analyses, wrote the manuscript, and shared scientific discussions.

## Conflict of Interest

The authors state that no competing financial interest exists.
